# Evaluating Heat Shock Proteins as Biomarkers for Vaginal Fungal Infections

**DOI:** 10.3390/jcm15082889

**Published:** 2026-04-10

**Authors:** Yazeed Albalawi, Mohammad Zubair

**Affiliations:** 1 Department of Obstetrics and Gynaecology, Faculty of Medicine, University of Tabuk, Tabuk 71491, Saudi Arabia; yalbalawi@ut.edu.sa; 2 Department of Medical Microbiology, Faculty of Medicine, University of Tabuk, Tabuk 71491, Saudi Arabia; 3 Molecular Microbiology and Infectious Disease Research Unit, University of Tabuk, Tabuk 71491, Saudi Arabia

**Keywords:** vulvovaginal candidiasis, heat shock proteins, HSP47, HSP90, biomarkers, inflammation, vaginal infection, ELISA

## Abstract

**Objective**: The purpose of this study was to determine the biological association between host-derived HSP47 and fungal-derived HSP90 in the context of vulvovaginal candidiasis (VVC) and to examine their relationships with clinical, inflammatory, and metabolic phenotypes in infected and healthy women. **Methods**: This study followed a six-month case–control design (February–July 2025) and was conducted at the University of Tabuk Hospital in Tabuk, Saudi Arabia. A total of 84 women aged 18–45 years were recruited, of which 42 were VVC-infected, and 42 were healthy controls. ELISA kits were used to test vaginal swabs for HSP47 and HSP90. Clinical, hematological, cytokine, and metabolic markers were also evaluated. Mann–Whitney U, Spearman correlation, and multiple linear regression tests were performed to analyze the data. **Results**: The levels of HSP47 and HSP90 were significantly higher among infected patients (2.29 ng/mL and 3341 ng/mL, respectively) when compared with controls (0.58 ng/mL and 1025.7 ng/mL; *p* < 0.001). Women who were infected were older (*p* = 0.02), but there were no significant differences in terms of BMI (*p* = 0.29). The levels of vitamin D and adiponectin were significantly decreased (*p* < 0.001), while pro-inflammatory cytokines (IL-6, TNF-α, IFN-γ, TGF-β, and IL-8) and WBC counts were higher compared to the control group. The hematology results were characterized by inflammation-related anemia and disturbed protein metabolism. The ROC analysis demonstrated good diagnostic performance, with an AUC of 1.0 in the case of HSP47 and 0.905 in the case of HSP90. In the case of the infected patients, the regression models were found to be weak (HSP90 R^2^ = 0.154; HSP47 R^2^ = 0.273), although HSP47 retained significant connections with IL-8 (*p* = 0.005) and IFN-γ (*p* = 0.028). **Conclusions**: High levels of HSP47 and HSP90 are observed in VVC, reflecting an epithelial stress response and fungal persistence. These HSPs have high diagnostic accuracy, which justifies their potential as biomarkers for the timely detection of VVC; they also have further implications as early biomarkers for prognostic and treatment monitoring support, despite the poor predictive models. This study has some limitations that must be addressed; in particular, the regression analyses failed to provide statistically significant predictive models, likely due to the limited sample size. In addition, the specificity of HSP90 and HSP47 for VVC in comparison with other vaginal infections was not evaluated.

## 1. Introduction

Vulvovaginal candidiasis (VVC), one of the most frequent infections with *Candida albicans*, occurs in women of reproductive age. At least one episode is experienced by up to 75% of women over the course of their lifetime, with around 5–10% experiencing recurrent VVC (RVVC)—characterized by three or more episodes per year [1,2,3]. Physical, emotional, and sexual health are affected by this chronic and highly incapacitating disease, resulting in serious morbidity [4]. Although commonly used, existing diagnostic tools for this purpose are not ideal, as they mostly depend on culture-based techniques and clinical assessment, which are time-consuming and may have limited sensitivity [5]. Therefore, fast, efficient biomarkers that can be used for diagnosis, risk stratification, and treatment planning are urgently needed.

*Candida albicans* is a commensal species in the human microbiota that may become a pathogen within the organism under specific drug, hormone, or immunosuppressive conditions [6]. The pathogenesis of VVC involves a complex of interactions between the host and pathogenic epithelial cells. When colonized, *Candida albicans* attaches to and invades the vaginal epithelium, leading to an inflammatory reaction, inducing immune cell recruitment and the secretion of pro-inflammatory cytokines [6]. This inflammatory environment leads to key clinical symptoms of VVC, including itching, burning, and abnormal discharge.

Heat shock proteins (HSPs) represent a group of highly conserved molecular chaperones that play important roles in cell homeostasis under stress conditions. HSP90 has been revealed as a central controller of virulence in *Candida albicans*, and can affect morphogenesis, biofilm formation, antifungal resistance, and so on [7]. *Candida albicans* extracellular HSP90 has been demonstrated to suppress immune responses in the host by inducing the NF-kB signaling pathway and pyroptosis in macrophages, thereby increasing the survival and virulence of pathogens [8]. These results emphasize the possibility of using HSP90 as a key biological marker in the context of VVC.

HSP47 is a collagen-specific chaperone that participates in the biosynthesis of collagen and remodeling of the extracellular matrix in the host. The HSP47 level has been shown to increase under different inflammatory states, indicating its possible use as a tissue biomarker of epithelial stress and immune regulation in VVC [9]. It has also been demonstrated that the expression of HSP47 is increased in response to transforming growth factor-beta (TGF-β1), a cytokine associated with fibrosis and tissue remodeling [10]. These data confirm the involvement of HSP47 in the pathophysiology of VVC, suggesting its potential utility as a biomarker.

Although the roles of fungal- and host-derived HSPs have been examined individually in VVC, few studies have examined their simultaneous expression in vaginal secretions. This research gap restricts knowledge about the interactions between the host and the pathogen during infection. As such, simultaneously studying fungal-derived HSP90 and host-derived HSP47 could provide a clearer picture of the associated host–pathogen interactions and provide further clues regarding the pathophysiology of VVC.

The two-biomarker strategy was chosen based on the complementary effects of HSP90 and HSP47, as well as their involvement in key aspects of virulence such as morphogenesis, antifungal resistance, epithelial stress, and immune regulation. Combining these biomarkers may provide an improved method for diagnosing and predicting VVC, allowing clinicians to diagnose infections earlier, anticipate the risk of a relapse, and adjust treatment methods more efficiently.

The study hypothesis is that a combination of host-derived HSP90 in vaginal secretions may act as a combined biomarker panel for the early diagnosis and prognosis of VVC and RVVC. The aims of this study were as follows:

- To determine the expression levels of HSP90 and HSP47 in vaginal secretions of women with VVC and RVVC.
- To determine the relationships between the levels of HSP90 and HSP47 and clinical outcomes such as the severity of symptoms and frequency of recurrence.
- To establish the diagnostic sensitivity and specificity of the HSP90/HSP47 biomarker panel, compared to conventional diagnostic techniques.

The validation of this two-biomarker (HSP90 and HSP47) panel may transform VVC testing practice, allowing practitioners to diagnose infections at an earlier phase and better determine the likelihood of recurrence. This would make timely and focused interventions easier, thus promoting a reduction in the disease burden and improving patient outcomes. Moreover, an understanding of the mechanistic roles of these biomarkers in the pathophysiology of VVC may guide the creation of new treatment options to control host–pathogen interactions. The integration of fungal- and host-derived biomarkers may help to fill the disconnect between pathogen biology and host responses, thereby resulting in more individualized and effective treatment plans, subsequently improving the quality of life of women with this common and yet not widely acknowledged disease.

## 2. Materials and Methods

### 2.1. Study Design and Setting

This case–control study was carried out in a hospital setting, with the aim of determining whether heat shock proteins (HSP47 and HSP90) present any significant biological associations and can serve as potential biomarkers for vulvovaginal candidiasis (VVC). This study was conducted in the University of Tabuk Hospital Clinic. The clinical examination and laboratory analyses were performed in the 6 months between February and July of 2025.

### 2.2. Sample Size

The sample size was calculated using the G* Power software (version 3.1.9.4) [9]. An a priori analysis was conducted to compare HSP47 and HSP90 expression levels between healthy and infected women using an independent samples *t*-test. Assuming a large effect size (Cohen’s d = 0.80), type I error (alpha = 0.05), and a statistical power of 0.95, the required minimum sample size was 84, with 42 in each group, ensuring adequate sensitivity to detect meaningful differences in biomarker expression.

### 2.3. Inclusion and Exclusion Criteria

For both groups, the inclusion criteria were as follows: females aged between 18 and 45 years old, who are not pregnant and have no history of systemic illness (diabetes or autoimmune disease), have not received recent antibiotics or antifungal therapy in the last 2 weeks, and have given informed consent. The exclusion criteria included pregnancy or lactation, the presence of bacterial or viral co-infections, under immunosuppressive therapy, a history of recurrent vulvovaginal candidiasis (at least 4 episodes/year), recent menstruation, and incomplete laboratory or clinical records.

### 2.4. Ethical Considerations

The study protocol was examined by the Local Research Ethics Committee of the Kingdom of Saudi Arabia within the National Research Ethics Committee (Approval ID: UT-716-451-2025). All participants signed informed consent before enrollment, and all procedures followed the principles of the Declaration of Helsinki to guarantee their safety and confidentiality.

### 2.5. Clinical Diagnosis

The diagnosis of infection was made through clinical and laboratory tests based on a thorough growth evaluation involving gynecological examination and laboratory confirmation. The first vaginal secretions that were studied under wet mount microscopy were prepared with 10% potassium hydroxide (KOH) (Merck, Rahway, NJ, USA), and budding yeast and pseudohyphae were identified. Gram staining was performed to establish the presence of fungal elements, with fungal culture carried out on Sabouraud Dextrose Agar (Oxoid, Hampshire, UK) at 37 °C and incubated for 48 h. Patients whose cultures were positive were considered the infected group, and asymptomatic women with negative cultures were regarded as controls.

### 2.6. Collection of Samples for HSP Analysis

Sterile Dacron swabs (Himedia India) were used to collect the vaginal secretions to be used as the collection of samples for HSP analysis. All swabs were placed on ice and soaked in 1 mL of phosphate-buffered saline (PBS, pH 7.4) (Merck, USA) and sent to the laboratory. To clear debris, samples were centrifuged at 3000 rpm for 10 min at 4 °C. The supernatant that was obtained was aliquoted and kept at −80 °C until further use.

### 2.7. HSP Quantification (HSP47 and HSP90)

The concentrations of HSP47 and HSP90 in the samples were measured using ELISA kits. HSP47 was quantified with the Human HSP47 ELISA Kit (Cloud-Clone Corp., Wuhan, China; Catalog No. SEA883Hu; detection range: 0.156–10 ng/mL; sensitivity: <0.059 ng/mL), while HSP90 was measured using the Human HSP90a ELISA Kit (Cloud-Clone Corp.; Catalog No. SEB906Hu; detection range: 0.312–20 ng/mL; sensitivity: <0.1 ng/mL). All protocols were followed according to the manufacturers’ instructions, and all samples were tested in triplicate to ensure reproducibility. Absorbance was measured at 450 nm using a BioTek ELx808 microplate reader (BioTek Instruments, Winooski, VT, USA), and concentrations were calculated based on standard calibration curves.

### 2.8. Cytokine Analysis

Cytokine profiling was also performed to complement the HSP analysis. The vaginal fluid supernatants were also examined for the levels of IL-6, IL-8, TNF-α, IFN-γ, TGF-β, and metabolic cytokines such as adiponectin and cathepsin D. High-sensitivity ELISA kits from R&D Systems (Minneapolis, MN, USA) were used for this purpose. The kit specifications are as follows: IL-6 (Catalog No. HS600B; detection range 0.156–10 pg/mL; sensitivity <0.09 pg/mL), IL-8 (Catalog No. D8000C; detection range 31.2–2000 pg/mL; sensitivity <7.5 pg/mL), and TNF-α (Catalog No. HSTA00E; detection range 0.5–32 pg/mL; sensitivity <0.106 pg/mL). These were performed in duplicate, and intra- and inter-assay variability was kept to less than 10% (as suggested by the manufacturer). Depending on the requirements of the kits, results are given in pg/mL or ng/mL.

### 2.9. Data Collection and Management

Clinical records and participant questionnaires were used to collect demographic and clinical data, including (but not limited to) age, body mass index (BMI), and self-reported vaginal discharge, pruritus, and burning sensation. A unique identifier was given to each participant to ensure confidentiality, and all data were stored in a password-protected database.

### 2.10. Statistical Analysis

The statistical analyses were conducted with the help of the IBM SPSS Statistics software (version 27; IBM Corp., Armonk, NY, USA). Descriptive statistics were calculated to describe demographic and clinical features (median and interquartile range). Non-normally distributed continuous variables were compared between groups using the Mann–Whitney U test, and the results are presented in the form of mean ranks and *p*-values. The correlations between HSP levels, cytokines, and clinical or laboratory markers were assessed using Spearman’s rank correlation coefficient. Multiple linear regression was performed to determine predictors of HSP47 and HSP90 expression, with cytokines and metabolic markers used as independent variables. The regression results are reported as standardized coefficients (b), R^2^ values, F-statistics, VIF, and standard error of the estimate. The diagnostic performance of HSP47 and HSP90 was also evaluated via Receiver Operating Characteristic (ROC) curve analysis, and the results are reported in terms of sensitivity, specificity, and area under the curve (AUC). A *p*-value less than 0.05 was considered statistically significant in the analysis.

## 3. Results

A total of 84 participants were included in this study, divided into two groups comprising 42 healthy and 42 infected individuals. This study involved examining the incidence of infection and health parameters among these two groups. Infected participants were older (median, 38 years) compared to healthy ones (median, 33 years), as shown in Table 1; however, BMI did not differ significantly between the groups (*p* = 0.299). HSP90 and HSP47 levels were significantly higher in infected subjects compared to healthy controls, indicating serious cellular stress (*p* < 0.001). Vitamin D levels were significantly decreased in infected cases (*p* < 0.001), which might be linked to an increased risk of infection. Metabolic changes were also observed, including a reduction in adiponectin (an anti-inflammatory marker) (18.65 ng/mL vs. 9.35 ng/mL). Immune activation was evident in the infected group, with significantly elevated inflammatory cytokines such as IL-6, IFN-γ, TNF-α, TGF-β, and IL-8 in this group (*p* < 0.001). Interestingly, LDL and VLDL concentrations were significantly lower (*p* < 0.001), possibly due to lipid redistribution caused by acute inflammation. The hematological results indicated increased WBC counts (5.3 × 10^9^/L vs. 7.9 × 10^9^/L), indicating immune activation, while Hb and RBC levels were reduced, suggesting anemia of inflammation. Additionally, serum total protein and albumin levels were decreased, whereas globulin levels increased in infected patients, indicating systemic inflammation and disrupted protein metabolism. Overall, these results suggest a complex physiological response to infection, involving immune system activation, metabolic alterations, and significant changes in inflammatory and nutritional markers.

Table 2 shows the results of the Mann–Whitney U test, revealing major discrepancies in various biomarkers between infected and healthy patients, demonstrating their potential utility. Infected patients were significantly older (*p* = 0.022), which means that the older individuals may be more susceptible to infection. The significantly (*p* ≤ 0.001) higher levels of HSP90 and HSP47 prove their importance as evidence for stress responses and potential as early markers. The concentrations of 25(OH)D and adiponectin were lower in the infected group (*p* < 0.001), indicating worse metabolic and immune control. A variety of inflammatory markers were significantly elevated (*p* < 0.001), including Cathepsin D, IL-6, IFN-γ, TNF-α, TGF-β, and IL-8, which is indicative of a systemic inflammatory response. A change in lipid profiles was also noted, with a significant decrease in HDL, while total cholesterol and VLDL were high, demonstrating dysregulated lipid metabolism in infected patients. The hematological values further evidenced an inflammatory process due to a high WBC count and decreased hemoglobin and RBCs (*p* < 0.001), which may culminate in anemia. In addition, the levels of total serum protein and albumin declined significantly, whereas the level of serum globulin increased (*p* < 0.01), further testifying to the existence of systemic inflammation. BMI and triglycerides also showed varying levels between the healthy and infected groups, although these differences were not significant.

**Figure 1:**
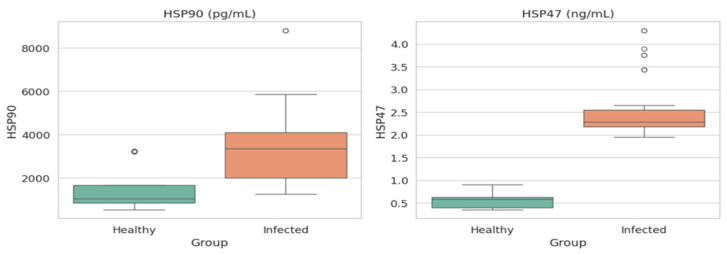
The distribution of HSP90 and HSP47 in healthy versus infected patients using box plots.

**Figure 2:**
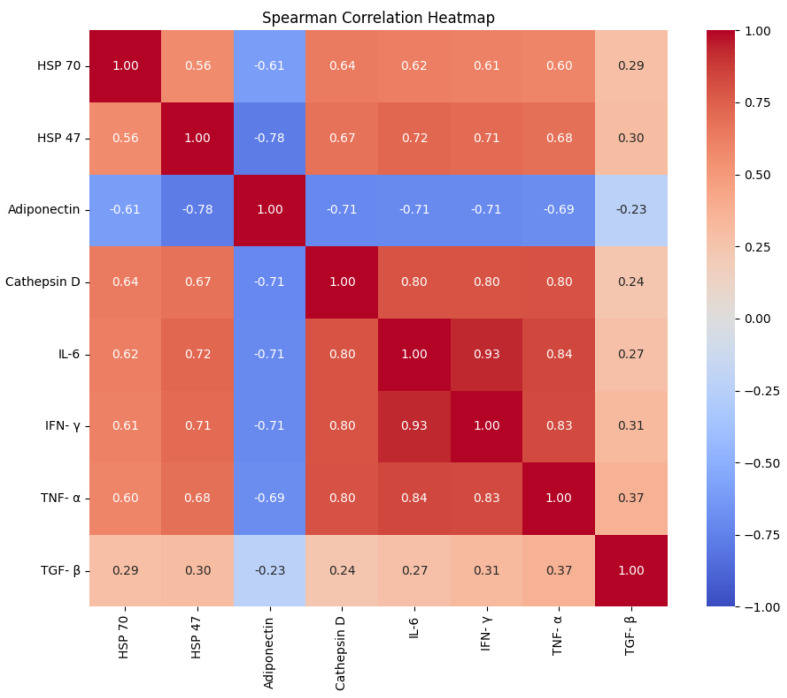
Spearman correlation heatmap illustrating the relationships between various biomarkers.

Figure 1 shows box plots comparing healthy individuals and infected patients in terms of the HSP90 and HSP47 level distribution. For HSP90, the box plot shows that the median and interquartile range for infected patients are significantly higher than those for healthy individuals, suggesting the utility of an elevated HSP90 level as a possible marker of infection, with a minor outlier in the healthy group indicating some variability. Conversely, the HSP47 box plot shows different medians and interquartile ranges for the healthy and treated groups. Infected patients exhibit slightly more variability and outliers than the healthy group, indicating that the differences between the two are less pronounced (Figure 1). The Spearman correlation heatmap in Figure 2 highlights the relationships between variables, with strong, positive correlations observed for HSP90 with itself (1.00) and moderate positive correlations with inflammatory factors such as IL-6, IFN-γ, and TNF-α (around 0.60–0.62), suggesting possible co-variation in these factors and their influence on the observed changes during infection. Overall, these findings suggest that HSP90 may be a more specific indicator of infection than HSP47 in the context of this study, with the relationships indicating a linked response to inflammation.

Multiple linear regression was carried out to determine the relationships between the inflammatory markers and the heat shock proteins HSP90 and HSP47 in infected patients (Table 3). In the case of HSP90, the regression results were not significant (F = 0.881, *p* = 0.532), and the model failed to capture fluctuations in HSP90 levels, reflecting only 15.4% of their variability (R^2^ = 0.154). Of all the predictors, adiponectin was the only variable with a positive response to HSP90 (B = 259.226) and approximate significance (*p* = 0.086); this finding may point out a potential trend to be considered in future research. Regarding the other variables, TGF-β and IL-8 showed moderate standardized low-significance beta values (beta = 0.526 and −0.539, respectively). In the case of HSP47, the regression model was modest but still not a good fit, capturing only 27.3% of the variance (R^2^ = 0.273, R = 0.523) and showing low significance (F = 1.825, *p* = 0.114). The constant value was significant (*p* < 0.001), which means that the baseline levels of HSP47 were high. TNF-α had a positive trend (β = 0.723, *p* = 0.107), while IL-8 showed a negative trend (β = −0.791, *p* = 0.109). None of the individual predictors was found to be statistically significant, indicating that they might also have relationships with the mechanism underlying the formation of HSP47 during infection. Although the models lacked statistical strength, the result suggests that adiponectin could affect the expression of HSP90, while TNF-α and IL-8 can affect the expression of HSP47; therefore, further experiments should be conducted to investigate the implications of these relationships with regard to the cellular stress response following infection. The multi-collinearity for the infected group model was assessed through VIF values, from which it was found that TNF-α, TGF-β, and IL-8 (VIF > 5) should be accepted as the remaining predictors in the model for prediction.

Table 4 shows the results of the multiple linear regression analyses performed to assess the associations of different inflammatory biomarkers with the expression of HSP90 and HSP47 in healthy individuals (Table 4). In the case of HSP90, the general linear regression analysis was not significant (*p* = 0.404), with a weak correlation (R = 0.425) and low explanatory potential (R^2^ = 0.180), indicating that the chosen biomarkers as a mixed group did not predict the HSP90 level significantly. One of the individual predictors was IL-8, for which a moderate inverse relationship (β = −87.583) of being significantly correlated with HSP90 (*p* = 0.036) was observed. All the other variables, including adiponectin, cathepsin D, IL-6, IFN-γ, TNF-α, and TGF-β, did not show significant relationships with HSP90 (all *p* > 0.05). Comparatively, the regression model for HSP47 was significant (*p* = 0.012), highly correlated (R = 0.625), and had moderate explanatory power (R^2^ = 0.391), explaining about 39% of the variation in the expression of HSP47. The significant positive predictor was IL-8 (B = 0.020, *p* = 0.005), while IFN-γ was found to have a significant negative correlation with HSP47 (B = −0.060, *p* = 0.028). The rest of the biomarkers—namely, adiponectin, cathepsin D, IL-6, TNF-α, and TGF-β—lacked statistically significant relationships (*p* 0.05), although IL-6 showed a borderline trend (*p* = 0.094). In addition, multi-collinearity was analyzed, and all predictor variables showed an acceptable window of acceptance (1 < VIF < 5), further supporting the model retaining all predictor variables in this analysis.

**Figure 3:**
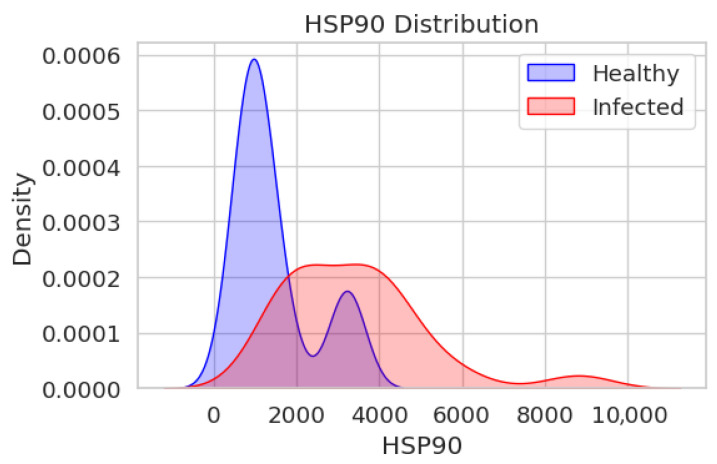
The distribution of HSP90 levels in healthy and infected individuals, presented as a Kernel Density Estimate (KDE) plot.

Figure 3 depicts the distribution of HSP90 levels for the two groups (healthy, blue; infected, red). A sharp peak can be observed for the healthy group at around 2000–3000 units HSP90, showing a focused distribution with the majority of values concentrated in this interval. The distribution in the infected group is more extended, with a peak at around 4000–5000 units HSP90, showing that there is a more varied range of HSP90 levels as well as a bias toward higher values when compared to the healthy group. The two distributions overlap slightly, especially between 2000 and 6000 HSP90 units; thus, despite there being an observable difference, there is an overlapping range of HSP90 levels between the two groups.

**Figure 4:**
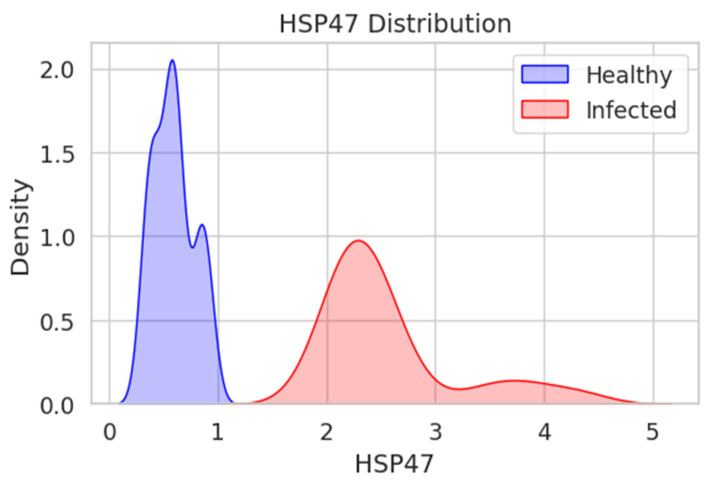
The distribution of HSP47 levels in healthy and infected individuals, presented as a Kernel Density Estimate (KDE) plot.

Similarly, Figure 4 shows the density distribution of HSP47 levels in the healthy and infected groups. The distribution for the healthy group presents a strong peak in the range of 1–2 HSP47 units that steeply declines on both sides, meaning that the distribution is tightly clustered. The peak for the infected group can be observed at 2–3 HSP47 units, with a more dispersed distribution toward 5 units. The two groups do not overlap very much, with a clear separation below 2 units, indicating a clear difference between healthy and infected individuals, where the latter shows a tendency to have higher HSP47 levels.

These distributions indicate that the HSP90 and HSP47 levels are not equal in the healthy and infected groups, with the infected group tending to be higher and more varied. The differences are more dominant in the case of HSP47, for which the distributions are more distinct.

**Figure 5:**
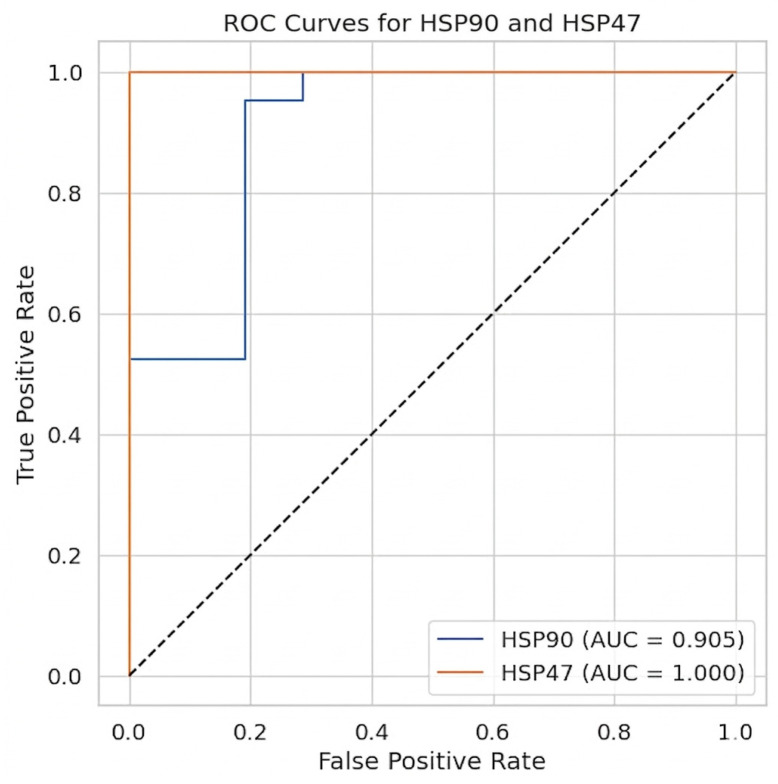
Receiver Operating Characteristic (ROC) curves for HSP90 and HSP47 (with AUCs of 0.905 and 1, respectively).

Figure 5 shows Receiver Operating Characteristic (ROC) curves for the HSP90 and HSP47 levels, which determine their effectiveness in separating between healthy and infected groups. The False Positive Rate appears on the x-axis, the True Positive Rate appears on the y-axis, and the diagonal dashed line represents random guessing (AUC = 0.5). An excellent discriminatory power is indicated by the blue curve of HSP90, with Area Under the Curve (AUC) of 0.905 (CI = 0.840–0.969, *p*-value 0.00); the curve steeply increases at the origin and, therefore, HSP90 is highly sensitive and yields comparatively few false positives. Notably, the orange curve of HSP47 attains an AUC of 1.000 (CI = 1.00–1.00, *p*-value 0.00), just below the upper-left corner of the curve, with a True Positive Rate of 1.0 at all False Positive Rates, indicating this marker as a potential diagnostic candidate due to its classification capability; however, it should be noted that this could be the consequence of using an idealized or very controlled dataset. Comprehensively, both HSP90 and HSP47 have good potential to serve as candidate biomarkers, of which it seems that HSP47 provides optimal discriminative ability according to the ROC analysis.

## 4. Discussion

The present study involved a comparative expression of host- and fungal-produced heat shock proteins (HSP47 and HSP90), inflammatory, and metabolic biomarkers between women with vulvovaginal candidiasis (VVC) and healthy controls. The results revealed dramatic upsurges of HSP90 and HSP47 in the infected patients, in addition to severe imbalances in immune mediators, hematology indices, lipids, and vitamin D metabolism. These results emphasize the complex pathophysiological nature of the response to VVC, as well as the potential of HSPs as prognostic biomarkers.

This study has some limitations that need to be addressed. The regression analyses failed to provide statistically significant predictive models, likely due to sample size limitations. Moreover, the specificity of HSP90 and HSP47 with respect to VVC over other vaginal infections was not assessed. Future research should include larger, multi-center cohorts to confirm the presented results and test the specificity of these HSPs with respect to various vaginal pathologies. Additionally, mechanistic studies are required to clarify the mechanisms connecting cytokine signals, metabolic dysregulation, and HSP expression in mucosal tissues. Furthermore, new therapeutic approaches to the management of RVVC may be developed through clinical trials examining HSP-targeted interventions. Finally, a comparative analysis between fungal and non-fungal vaginal infections (with the former responsible for one-third of VVC cases) is expected to help in further clarifying the underlying mechanisms, thus potentially broadening the scope of our findings.

The increased HSP90 levels observed in infected patients are consistent with the existing literature on its prominent role in *Candida albicans*-related pathogenesis. HSP90 promotes antifungal resistance by stabilizing main signaling proteins, implying that its elevation in VVC represents active invasion and resistance to drugs [11]. Furthermore, Fang et al. [8] demonstrated that extracellular HSP90 released by *Candida albicans* promotes virulence through regulation of NF-kB signaling and induction of macrophage pyroptosis—mechanisms that lead to a persistent inflammatory response and compromised immune ability. The positive correlations between HSP90 and a range of inflammatory cytokines (including IL-6, IFN-γ, and TNF-α) in the present study lend credibility to the mechanistic hypothesis that fungal HSP90 amplifies mucosal inflammation by modulating host immune pathways.

While HSP90 has been thoroughly studied in the context of fungal biology, HSP47 has not been assessed within the environment of VVC before, making this study one of the first to assess its relevance. HSP47 is a collagen-specific chaperone involved in extracellular matrix remodeling, which is upregulated when epithelial cells are stressed and fibrosis occurs [9]. The high HSP47 levels observed in the infected patients in the present study may indicate damage to and remodeling of the mucosal area of the host due to chronic inflammation. This result aligns with previous studies in which mucosal HSP expression—especially HSP60 and HSP70—was higher in women with periodic VVC [12]. Moreover, pregnancy-associated changes in vaginal microbial composition have been indicated to alter the epithelial expression of HSP70 and autophagy [13], indicating that host HSPs act as stress-sensitive microbiome imbalance sensors. The findings of the present study extend this idea to HSP47, which shows potential as a novel biomarker of epithelial integrity in fungal infections.

The infected group had high levels of inflammatory cytokines, such as IL-6, IFN-γ, TNF-α, IL-8, and TGF-β, which have also been previously reported in relation to a high pro-inflammatory response in VVC [14]. IL-6 and TNF-α are critical mediators of neutrophil recruitment and epithelial damage during VVC, and thus play a role in symptomatic disease, even though the dissemination of fungi in the body may occur without any signs [15]. The regression models demonstrated that TNF-α and IL-8 could mediate HSP47 expression, whereas adiponectin mediated the HSP90 concentration. These associations, although not statistically significant, indicate possible interactions between immune activation and the regulation of stress proteins. Pro-inflammatory cytokines may be a driver of epithelial stress responses leading to increased HSP47, while metabolic regulators such as adiponectin—which have anti-inflammatory functions—may dampen fungal stress responses, as reflected in HSP90 levels.

It is significant that a decrease in the vitamin D level is commonly observed in infected individuals; notably, vitamin D is an important immunomodulatory molecule with effects on both the innate and adaptive immunity. Vulvar recurrent VVC has been linked to low serum 25(OH)D, which may contribute to poor epithelial barrier and low antimicrobial peptide generation [16,17]. Similarly, the lower adiponectin levels in the infected group indicate impaired anti-inflammatory control. It has been reported that adiponectin reduces pro-inflammatory cytokines and epithelial healing, which implies that a decrease in its level may worsen infection-related inflammation [18]. Collectively, these data indicate a role of metabolic dysregulation in the risk of developing VVC through impairment of mucosal defense against VVC.

The increase in the WBC count and decreases in hemoglobin and RBC counts indicate anemia of inflammation, another typical host response to chronic infection [19]. In addition, decreased serum total protein and albumin, accompanied by an increase in globulins, is a sign of systemic inflammation and acute-phase response. Lipid digestion was also impaired, resulting in lowered HDL and modified LDL/VLDL. These results reflect the tendencies of systemic fungal infections, in which the redistribution of lipids due to inflammation promotes the activation of immune cells at the cost of circulating lipoproteins [19].

The study of HSP47 and HSP90 as HSP infection biomarkers has significant clinical significance regarding the diagnosis and treatment of vulvovaginal candidiasis (VVC). The ROC analysis demonstrated that HSP47 has superior discriminatory power (AUC = 1.0), and HSP90 has significant diagnostic power (AUC = 0.905); hence, their combination may bring about great improvements in diagnostic accuracy. These biomarkers have several possible clinical applications; for example, the measurement of HSP90 and HSP47 in vaginal secretions may provide a quick and objective method to differentiate VVC from other types of vaginitis, thus reducing the delays caused by traditional culture-based methods. Prognostically, high HSP47 levels could be an indicator of epithelial stress and a predictor of recurrence, while continuous high levels of HSP90 might be an indication of fungal persistence and antifungal resistance. Furthermore, the serial monitoring of these biomarkers in patients under treatment may be helpful to clinicians in terms of assessing therapeutic responses and detecting possible relapse, thereby offering an opportunity to create individualized treatment plans.

## 5. Conclusions

In conclusion, this study presented novel evidence that the levels of fungal-derived HSP90 and host-derived HSP47, representing different but complementary facets of infection biology, are highly increased in women with VVC. Due to their higher biological association, they have significant diagnostic potential in terms of their translational use as biomarkers in early diagnostics, prognosis, and treatment monitoring. HSPs hold promise for promoting the individualized management of vulvovaginal candidiasis, particularly through the modulation of the pathogen virulence process and host stress responses.

## Figures and Tables

**Table 1:** Descriptive statistics of clinical, biochemical, and inflammatory parameters in healthy individuals and infected patients.

	Patient Category		*p*-Value
	Healthy Individuals	Infected Patients	
	Median (IQR)	Median (IQR)	
Age	33 (27–40)	38 (36–40)	0.02
Body Mass Index (BMI)	22.77 (21.50–23.01)	21.05 (17.96–23.62)	0.299
Heat Shock Protein 90 (HSP90)	1025.7 (835.1–1653.6)	3341.1 (1984.5–4103.5)	<0.001
Heat Shock Protein 47 (HSP47)	0.58 (0.39–0.63)	2.29 (2.18–2.57)	<0.001
25-Hydroxyvitamin D (Vitamin D status indicator) (25(OH)D)	49.8 (41.2–58.4)	23.3 (16.0–33.0)	<0.001
Adiponectin	18.65 (15.8–21.4)	9.35 (7.8–10.6)	<0.001
Cathepsin D	140.7 (130.0–149.9)	513.9 (342.6–563.2)	<0.001
Interleukin-6 (IL-6)	2.8 (2.4–3.7)	28.3 (22.1–39.0)	<0.001
Interferon Gamma (IFN-γ)	5.5 (4.1–6.8)	43.3 (31.2–59.3)	<0.001
Tumor Necrosis Factor alpha (TNF-α)	2.8 (2.1–3.7)	36.1 (25.8–49.1)	<0.001
Transforming Growth Factor beta (TGF-β)	4.0 (3.1–4.5)	4.9 (3.7–6.1)	0.001
Interleukin-8 (pro-inflammatory chemokine)	19.5 (15.8–24.5)	43.0 (31.7–55.1)	<0.001
Total Cholesterol (mg/dL)	116 (110–133)	133 (130–135)	<0.001
Triglycerides (mg/dL)	89 (66–123)	95 (78–95)	0.438
High-Density Lipoprotein Cholesterol	54 (50–58)	34 (33–34)	<0.001
Low-Density Lipoprotein Cholesterol	94 (80–110)	81 (81–82)	0.001
Very Low-Density Lipoprotein Cholesterol	25 (20–30)	19 (16–19)	<0.001
White Blood Cell Count (×10^9^/L) WBC	5.3 (4.7–6.0)	7.9 (6.9–10.4)	<0.001
Hemoglobin (g/dL) Hb	13.7 (12.9–14.5)	11.0 (9.5–11.7)	<0.001
Red Blood Cell Count (×10^6^/μL) RBC	4.55 (4.30–4.80)	3.89 (3.40–4.40)	<0.001
Total Serum Proteins (g/dL) (TSP gms%)	7.0 (6.8–7.3)	6.6 (6.1–7.3)	0.007
Serum Albumin (g/dL) (SA gms%)	4.2 (4.0–4.4)	3.5 (3.0–3.8)	<0.001
Serum Globulin (g/dL) (SG gms%)	2.7 (2.5–3.0)	3.4 (3.0–3.7)	0.001

**Table 2:** Comparison of clinical and biochemical parameters between healthy and infected groups using the Mann–Whitney U test.

Variable	Mean Rank (Healthy)	Mean Rank (Infected)	U	Z	*p*-Value
Age	36.43	48.57	627	−2.292	0.022
BMI	45.26	39.74	766	−1.038	0.299
HSP90	25.5	59.5	168	−6.394	<0.001
HSP47	21.5	63.5	0	−7.898	<0.001
25(OH)D	60.92	24.08	108.5	−6.921	<0.001
Adiponectin	63.48	21.52	1	−7.883	<0.001
Cathepsin D	26.7	56.5	0	−4.604	<0.001
IL-6	31.8	55.5	0	−6.453	<0.001
IFN-γ	33.4	67.2	0	−8.538	<0.001
TNF-α	21.5	63.5	0	−7.891	<0.001
TGF-β	34.02	50.98	526	−3.186	0.001
IL-8	22.89	62.11	58.5	−7.368	<0.001
Cholesterol	31.38	53.62	415	−4.207	<0.001
Triglycerides	40.45	44.55	796	−0.775	0.438
HDL Cholesterol	63.42	21.58	3.5	−7.922	<0.001
LDL Cholesterol	50.98	34.02	526	−3.214	0.001
VLDL Cholesterol	55.67	29.33	329	−4.997	<0.001
WBC	24.73	59.11	135.5	−6.539	<0.001
Hb	62.8	22.2	29.5	−7.63	<0.001
RBC	56.26	28.74	304	−5.177	<0.001
TSP gms%	49.62	35.38	583	−2.681	0.007
SA gms%	60.36	24.64	132	−6.73	<0.001
SG gms%	31.46	53.54	418.5	−4.156	<0.001

**Table 3:** Regression analysis of biomarkers with respect to HSP90 and HSP47 expression in infected cases.

Variable (Unit)	HSP90 (Infected)					HSP47 (Infected)					VIF
	B	Std. Error	Beta	t	*p*-Value	B	Std. Error	Beta	t	*p*-Value	
**(Constant)** —	−84.97	1878.822	—	−0.045	0.964	4.303	0.62	—	6.937	<0.001	-
**Adiponectin** (ng/mL)	259.226	146.362	0.301	1.771	0.086	−0.066	0.048	−0.215	−1.362	0.182	1.164
**Cathepsin D** (ng/mL)	1.266	2	0.104	0.633	0.531	−0.001	0.001	−0.125	−0.826	0.415	1.079
**IL-6** (pg/mL)	30.83	43.336	0.167	0.711	0.482	0	0.014	−0.002	−0.008	0.993	2.203
**IFN-γ** (pg/mL)	−22.093	27.158	−0.195	−0.813	0.422	−0.006	0.009	−0.142	−0.641	0.526	2.299
**TNF-α** (pg/mL)	16.195	68.578	0.111	0.236	0.815	0.038	0.023	0.723	1.657	0.107	8.917
**TGF-β** (pg/mL)	642.692	446.248	0.526	1.44	0.159	−0.071	0.147	−0.162	−0.48	0.634	5.354
**IL-8** (pg/mL)	−72.864	69.976	−0.539	−1.041	0.305	−0.038	0.023	−0.791	−1.648	0.109	10.781

**Table 4:** Regression analysis of biomarkers with respect to HSP90 and HSP47 expression in healthy individuals.

Variable (Unit)	HSP90 (Healthy)					HSP47 (Healthy)					VIF
	B	Std. Error	Beta	t	*p*-Value	B	Std. Error	Beta	t	*p*-Value	
**(Constant)** —	1920.988	2593.969	—	0.741	0.464	0.811	0.44	—	1.843	0.074	
**Adiponectin** (ng/mL)	−70.731	51.184	−0.267	−1.382	0.176	0.001	0.009	0.024	0.147	0.884	1.553
**Cathepsin D** (ng/mL)	16.819	15.863	0.211	1.06	0.296	−0.005	0.003	−0.287	−1.677	0.103	1.638
**IL-6** (pg/mL)	129.987	365.827	0.105	0.355	0.725	0.107	0.062	0.44	1.721	0.094	3.650
**IFN-γ** (pg/mL)	61.905	154.992	0.113	0.399	0.692	−0.06	0.026	−0.563	−2.299	0.028	3.345
**TNF-α** (pg/mL)	−33.385	347.074	−0.03	−0.096	0.924	−0.092	0.059	−0.421	−1.556	0.129	4.092
**TGF-β** (pg/mL)	−102.687	299.855	−0.094	−0.342	0.734	0.068	0.051	0.313	1.328	0.193	3.097
**IL-8** (pg/mL)	−87.583	40.163	−0.551	−2.181	0.036	0.02	0.007	0.654	3.005	0.005	2.644

## Data Availability

Data are available from the corresponding author upon reasonable request.
